# Determining biological variation of serum parathyroid hormone in healthy adults

**DOI:** 10.11613/BM.2019.030702

**Published:** 2019-08-05

**Authors:** Müjgan Ercan, Emiş Deniz Akbulut, Esin Avcı, Çiğdem Yücel, Esra Fırat Oğuz, Turan Turhan, Muhittin Serdar

**Affiliations:** 1 Faculty of Medicine, Department of Biochemistry, Harran University, Şanlıurfa, Turkey; 2 Biochemistry Laboratory, University of Health Sciences, Ankara Child Health and Diseases Hematology Oncology Training and Research Hospital, Ankara, Turkey; 3 Faculty of Medicine, Department of Biochemistry, Pamukkale University, Denizli, Turkey; 4 Biochemistry Laboratory, Ankara Numune Training and Research Hospital, Ankara, Turkey; 5 Faculty of Medicine, Department of Biochemistry, Acıbadem University, İstanbul, Turkey

**Keywords:** biological variation, individuality, reference change value, PTH, quality specification

## Abstract

**Introduction:**

Measurement of parathyroid hormone (PTH) is essential in the investigation and management of calcium metabolism disorders. To assess the significance of any assay result when clinical decision making biological variation (BV) of the measurand must be taken into consideration. The aim of the present study is determining the BV parameters for serum PTH.

**Materials and methods:**

Blood samples were taken at weekly intervals from 20 healthy subjects for ten weeks in this prospective BV study. Serum “intact PTH” concentrations were measured with electrochemiluminescence method. Biological variation parameters were estimated using the approach proposed by Fraser.

**Results:**

The values of within-subject biological variation (CV_I_), between-subject biological variation (CV_G_), analytical variation (CV_A_), reference change value (RCV) and individuality index (II) for serum PTH were 21.1%, 24.9%, 3.8%, 59.4% and 0.8%, respectively. Within-subject biological variation and CV_G_ were also determined according to gender separately; 18.5% and 24.0%; 26.2% and 18.6% for male and female, respectively. Calculated desirable precision and bias goals were < 10.6% and < 6.3%, respectively.

**Conclusion:**

This study may contribute to BV data on serum PTH as it includes a sufficient number of volunteers from both genders over an acceptable period of time. We do not recommend the usage of population-based reference intervals for serum PTH concentrations. Reference change value may be helpful for the evaluation of serial serum PTH results. Nonetheless, evaluation of data according to gender is necessary when setting analytical performance specifications.

## Introduction

Parathyroid hormone (PTH) is a large polypeptide hormone initially synthesized as a pre-proprotein which later undergoes cleavages to generate the biologically active fragment containing 84 amino acid (*1*). It is stored in the parathyroid glands and secreted into circulation to regulate calcium and phosphate homeostasis through interacting with its receptors localised on the target cells in renal tubules, bone and intestine (*2*-*4*). Measurement of PTH has a major effect on the accurate diagnosis and treatment of related metabolic disorders (*5*, *6*). Interpretation of PTH test results is always a challenge for both physicians and laboratory specialists. There are many factors affecting PTH results such as diurnal and seasonal rhythms and poor standardization of sample collection and differences in antibodies used by different methods. All these variations should be kept in mind to obtain reliable PTH results (*7*-*10*).

In the clinical laboratory, production and applications of data on biological variation (BV) data is essential in order to improve the quality of laboratory testing process (*11*, *12*). For this purpose, the main measures derived from BV data and used in the laboratory practice are determination of analytical goals, evaluation of population based reference values for convenience and assessment of the significant difference between serial results belonging to the same individual (*13*-*15*). Components of BV are: i) within-subject biological variation (CV_I_) which is defined as the random fluctuations around a set point within the same individual and ii) between-subject biological variation (CV_G_), which is defined as the variations occurring due to different equilibrium points of different individuals. Calculation of CV_I_ and CV_G_ values is needed for the definition of desirable specifications (*11*, *12*, *16*).

Presentation of new data on CV_I_ and CV_G_ values, setting goals for accuracy, precision and total acceptable error derived from these BV estimates for an analyte will contribute to the literature and clinical laboratory practice in terms of process improvement. This is the first study in Turkey related to the biological variation of PTH. To the best of our knowledge, there are only three pre-existing reports related with BV of PTH in literature which were conducted in the United Kingdom (UK) (*17*-*19*). In previous studies, plasma was the specimen type while we used serum samples as extensively done in our country. We also carried on sampling for a longer period of time (10 weeks) than the other reports. Additionally, PTH data stratified according to gender was missing in the literature.

The aim of the present study is to determine the BV of serum PTH. We were also intended to contribute to clinical BV applications used in health care systems.

## Materials and methods

### Subjects

This prospective study was conducted at Ankara Numune Training and Research Hospital Biochemistry Laboratory, Ankara, Turkey. Twenty healthy volunteers with 10 male (median age 34 years, range: 25-54) and 10 female (median age 32 years, range: 23-40) subjects were included. The participants were in completely healthy status and they did not use any kind of medication or herbal supplement. The exclusion criteria were having concomitant autoimmune or autoinflammatory disease, acute or chronic infection, malignancy, systemic diseases such as diabetes mellitus and heart failure, pregnancy or being in the postpartum 6 months, kidney disease, undergone parathyroidectomy or bone disease. The participants were advised to maintain their normal lifestyles while the study was conducted. The study protocol was approved by the Ethics Committee of Ankara Numune Training and Research Hospital (Ref number: 1529/2017) and written informed consent was obtained from each participant.

### Methods

Peripheral venous blood specimens were drawn according to the recommendations of the Clinical Laboratory Standards Institute (CLSI), Document GP41 (Collection of Diagnostic Venous Blood; Approved guideline, 7^th^ *ed*.) in order to minimise preanalytical variability. Subjects underwent phlebotomy between 9-10 in the morning after a fasting period of 8 hours by the same phlebotomist on the same day of the week for 10 consecutive weeks between April 2017 and June 2017. The samples were collected in serum vacuum tubes containing separator gel (volume of 5 mL) BD Vacutainer SST II advance (Ref number: 8260910) (Becton, Dickinson and Company Franklin Lakes, NJ, USA) and sera were obtained after centrifugation at 1500xg for 10 minutes. Afterwards, serum samples were aliquoted into Eppendorf tubes and kept at - 80°C until analysis. Serum concentrations of calcium and phosphate were analyzed on biochemistry autoanalyzer Cobas c702 (Roche Diagnostics, Mannheim, Germany) and PTH concentrations were analyzed on Cobas e601 electrochemiluminescence immunoassay system (Roche Diagnostics, Mannheim, Germany) by 2^nd^ generation Elecsys PTH assay.

All samples belonging to the same volunteer were assayed in the same batch for minimising inter-batch analytical variation. All samples and internal quality control materials were analyzed in duplicate. We conducted an internal quality control process using 2 levels of PreciControl Varia (Ref number: 05618860) (Roche Diagnostics, Mannheim, Germany). Internal quality control process was within acceptable ranges during the study period. The same lots of calibrators, reagents and quality control materials were used during the study and all analyses were performed by the same analyst.

### Statistical analysis

Prior to analysis, outliers were identified and removed (Bartlett and Cochran tests). Subsequently, confirmation of the outliers was done by visually inspection a graph of data (scatter plot diagram). The Anderson-Darling test was used to check the normality of within- and between-subject data. Data were expressed as the mean ± standard deviation for normally distributed data. The differences between mean values in female and male groups were tested using Student’s *t*-tests. The values P < 0.05 were considered statistically significant. To calculate CV_I_, CV_G_ and analytical variation (CV_A_) nested analysis of variance (nested ANOVA) was used.

Analytical variation was calculated from the duplicate results of each sample. Standard deviation (SD), CV_A_, CV_I_, CV_G_, individuality index (II) and reference change value (RCV) were calculated according to the formulas described by Fraser and Harris as follows (*20*):

SD_A_^2^ = (∑d^2^/2n) and CV_A_ = (SD_A_/Mean) x 100

CV_I_ = (CV_TI_^2^ – CV_A_^2^)^1/2^

CV_G_ = (CV_T_^2^ – CV_I_^2^ – CV_A_^2^)^1/2^

II = CV_I_ / CV_G_

RCV = 2^1/2^ x Z x (CV_A_^2^ + CV_I_^2^)^1/2^,

where SD_A_ represents the analytical standard deviation; d the difference between duplicates; n the number of paired result; CV_TI_ the total within subject variation, and Z the probability selected for statistical significance (Z = 1.96 at 95% confidence interval, CI).

Biological variation estimates were used to calculate analytical performance specifications (APS) for analytical imprecision, for analytical bias and for total allowable error. Data analysis was performed using Analyse-it for Microsoft Excel 4.0 (Analyse-it Software Ltd., Leeds, UK) and XLSTAT^®^ software (Addinsoft, Paris, France).

## Results

Distribution of serum calcium (2.3 ± 0.1 mmol/L) and inorganic phosphate (1.2 ± 0.2 mmol/L) concentrations were given in Table 1. We included 371 measurements for all calculations from the 400 results. Total of outliers was 7.25%.

**Table 1:** Serum concentrations of the measured analytes

**Subjects** **(population based reference intervals)**	**All group** **(N = 20)**	**Male** **(N = 10)**	**Female** **(N = 10)**	**P**
PTH (15-65 ng/L)	34.6 ± 10.9	38.2 ± 11.7	31.1 ± 8.8	< 0.001
Calcium (2.1-2.5 mmol/L)	2.3 ± 0.1	2.4 ± 0.1	2.3 ± 0.2	0.500
Inorganic phosphate (0.8-1.5 mmol/L)	1.2 ± 0.2	1.3 ± 0.1	1.1 ± 0.2	0.200
Data are presented as mean ± standard deviation. PTH - parathyroid hormone. P < 0.05 was considered statistically significant.				

**Figure 1:**
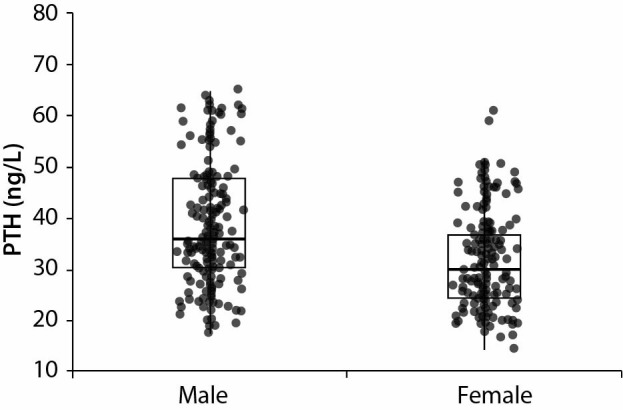
Distribution of iPTH concentration according to gender

Table 2 lists CV_A_, CV_I_, CV_G_ with the corresponding 95% coefficients of variation (CIs), II and RCV for PTH. There were no significant differences in 95% CIs for CV_I_ and CV_G_ estimated between females and males. Serum PTH distributions for both genders were given as box plot graph in Figure 1.

**Table 2:** Variance components for PTH derived from BV data

	**Male**	**Female**	**All group**
CV_A,_ % (95%CI)		3.8 (3.3-4.5)	
CV_I_, % (95%CI)	18.5 (15.8-21.5)	24.0 (21.5-27.6)	21.1 (19.4-23.2)
CV_G_, %(95%CI)	26.2 (17.5-48.7)	18.6 (11.6-35.8)	24.9 (18.4-37)
II	0.7	1.2	0.8
RCV	52.3	67.3	59.4
CV_A_ was calculated using the duplicate results of participants. CV_A_ - analytical coefficient of variation. CI - coefficient of variation. CV_I_ - within-subject biological variation. CV_G_ - between-subject biological variation. II - individuality index. RCV - reference change value			

**Figure 2:**
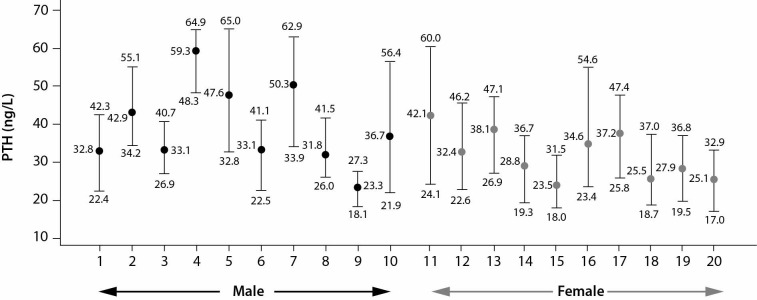
Mean with range (minimum-maximum) of iPTH test for individuals based on weekly samplings for 10 weeks

The mean values of PTH results for two genders were shown in Figure 2. Three level model for APS including minimum, desirable and optimal goals for precision, bias and total error for PTH derived from the biological data for all group were shown in Table 3.

**Table 3:** Analytical performance specifications for PTH measurement derived from our data on BV for all group

**Quality level**	**Precision**	**Bias**	**TE_a_**
Optimal performance, %	< 5.3	< 3.1	< 11.8
Desirable performance, %	< 10.6	< 6.3	< 23.7
Minimal performance, %	< 15.8	< 9.4	< 35.5
TE_a_ - total allowable error. CV_A_ - analytical coefficient of variation. CV_I_ - within-subject biological variation. CV_G_ - between-subject biological variation. Optimal performance: Imprecision CV_A_ < 0.25 CV_I_, Bias < 0.125 (CV_A_^2^ + CV_G_^2^)^1/2^, TEa < 0.125 (CV_A_^2^ + CV_G_^2^)^1/2^ + 1.65 (0.25 CV_I_). Desirable performance: Imprecision CV_A_ < 0.50 CV_I_, Bias < 0.250 (CV_A_^2^ + CV_G_^2^)^1/2^, TEa < 0.250 (CV_A_^2^ + CV_G_^2^)^1/2^ + 1.65 (0.5 CV_I_). Minimal performance: Imprecision CV_A_ < 0.75 CV_I_, Bias < 0.375 (CV_A_^2^ + CV_G_^2^)^1/2^, TEa < 0.375 (CV_A_^2^ + CV_G_^2^)^1/2^ + 1.65 (0.75 CV_I_).			

## Discussion

The present study revealed that PTH has high within and between individual biological variations with low individuality index in healthy adults. We also detected that CV_ı_ and CV_G_ values were similar for both gender. In the study by Ankrah-Tetteh *et al*. the results were similar to our findings, as CV_I_ and CV_G_ were 25.9 and 23.8, respectively (*17*). Viljoen *et al.* planned their study on twenty healthy volunteers and revealed that CV_I_ and CV_G_ for PTH were 25.3% and 43.4%, respectively (*18*). While the CV_I_ result was similar to our finding, the CV_G_ was higher than our result. Gardham *et al.* reported a value of 19.2% for CV_I_ from a group including 12 healthy participants (*19*).

This is the first report providing BV parameters of PTH with 95% CIs separately for both genders and all subjects. The CIs of the CV_I_ and CV_G_ values were not reported previously, rendering a comparison of BV data difficult between studies. Lack of gender-stratification may limit the utility of BV estimates. In this study, APS were calculated using data from all participants since 95% CIs of CV_I_ and CV_G_ values according to genders overlapped.

For estimates of components of biological variation to be valid, the analytical component of variation has to be less than half of the intraindividual variation (*21*). Results of the other previous studies and our study are less than half of CV_I_. In order to achieve and maintain quality together with the generation of reliable test results in the clinical laboratory performance targets are required (*11*, *22*). The desirable precision, bias and total allowable error for serum PTH determination was < 10.6%, < 6.3% and < 23.7%, respectively, according to the BV data obtained in this study.

In the clinical practice interpretation of a test result’s significance according to the reference interval may not ensure an accurate evaluation all the time. Individuality index is the preferred marker used to check the convenience of reference interval for result evaluation. When II of a test is less than 0.6, the distribution of the values of any individual is expected to be presented only in a small portion of the interval which means significant individuality. In such a situation, classical reference values will not be useful for the evaluation of results. If II > 1.4, the use of reference range is acceptable but if II < 1.0 it is not appropriate (*22*). According to our results mean PTH concentration in females was lower than that of males (P < 0.001). However, 95% CIs showed the insignificant difference between genders. CV_I_ was higher among females while CV_G_ was higher among males. Calculated II values as 0.8 for all participants; 0.7 for males and 1.2 for females. These values indicated that PTH results should not be evaluated according to reference intervals especially for men mostly depending on the relatively smaller intraindividual variation which meant that any small change between the consecutive measurements, while the results were still in the reference interval, may be reflecting a significant change in the clinical course. Viljoen *et al.* reported II 0.5 in their study for all genders (*18*). So their findings support ours that PTH results should be evaluated in terms of clinical outcomes, gender and other factors affecting biological variation. Reference change value is another related measure derived from BV data. The difference between consecutive measurements of an individual can be detected before the reference interval is exceeded via using RCV. For many analytes utility of RCV concept may be more helpful in the clinical decision-making process rather than the population-based normal interval as a reference interval (*14*). Calculated RCVs in our study for all individuals, females and males in 95% confidence interval were 59.4%, 67.3% and 52.3%, respectively. With a better analytical precision probability of changes being meaningful will be higher which means a more sensitive RCV.

Hormone parameters are hard to interpret because of poor standardization and harmonization among methodologies. This challenging situation may also be leading to a discrepancy between BV estimates when different generations of PTH assay and immunoassay systems are used. All of the previous studies used second generation “intact” PTH assay on various systems (*17*-*19*). In the present study, PTH concentrations were analyzed on Cobas e601 electrochemiluminescence immunoassay system (Roche Diagnostics, Mannheim, Germany) by 2*^nd^* generation Elecsys PTH assay.

In this study, serum was the preferred specimen for PTH just like the routine practice in our laboratory. Ankrah-Tetteh *et al*. also used serum specimens while Viljoen *et al.* and Gardham *et al.* conducted their study on plasma samples for PTH examinations. In the daily laboratory practice using serum has an advantage like making the analyses of calcium and phosphate possible from the same sample in addition to PTH, whereas plasma is supposed to have better stability for PTH (*7*).

According to Fraser and Harris components of variation can be assessed from a relatively small number of specimens collected from a small group of subjects over a reasonably short period of time (*20*). To our knowledge, only Røraas *et al.* have reported about the effect of subject number on the reliability of biological variation studies and calculated power of a variety of experimental designs with varying ratios between the analytical imprecision and the within-subject variation (*23*). Accordingly, our study’s power is 1.00. In a review by Ricos *et al.* the number of subjects included and samples were taken stated to be not very important, but at least 10 subjects and 5 samples were recommended (*13*). In the light of all these recommendations, samples collected from 20 participants throughout 10 consecutive weeks may be accepted as a quite sufficient sample size for a BV study.

One limitation of our study is the lack of knowledge about vitamin D status of the participants. The correlation between serum vitamin D and serum PTH concentrations is a well-known relationship. But we included healthy volunteers in the study so vitamin D deficiency may not be clinically overt. Finally, variation sources like sample type are needed to be considered while interpreting PTH results. According to the results of this study, we do not recommend the use of population-based reference intervals, furthermore RCV may be helpful in the evaluation of consecutive results of serum PTH measurement.

In conclusion, this study may contribute to BV data on serum PTH as it includes a sufficient number of volunteers from both genders over an acceptable period of time.
